# Isolation, Identification, and Antimicrobial Susceptibilities of Bacteria from the Conjunctival Sacs of Dogs with Bacterial Conjunctivitis in Different Regions of Wuhan, China

**DOI:** 10.3390/vetsci12010021

**Published:** 2025-01-06

**Authors:** Yuxin Li, Yinan Wang, Xin Gao, Lihong Luo, Bohan Zhang, Xiao Wang, Jing Li, Ruijia Wu, Lixin He, Wenxuan Li, Changwei Qiu

**Affiliations:** Department of Clinical Veterinary Medicine, College of Veterinary Medicine, Huazhong Agricultural University, Wuhan 430070, China; li-yuxin@webmail.hzau.edu.cn (Y.L.); wangyinan12@webmail.hzau.edu.cn (Y.W.); gaoxin07@webmail.hzau.edu.cn (X.G.); lihongluo@webmail.hzau.edu.cn (L.L.); 2022302110218@webmail.hzau.edu.cn (B.Z.); wang-xiao@webmail.hzau.edu.cn (X.W.); lijingn@webmail.hzau.edu.cn (J.L.); 2023302120144@webmail.hzau.edu.cn (R.W.); hlx2020@webmail.hzau.edu.cn (L.H.); liwenxuan@webmail.hzau.edu.cn (W.L.)

**Keywords:** dogs, bacterial conjunctivitis, aminoglycoside antibiotics, antimicrobial susceptibility test, resistance genes

## Abstract

In order to investigate the bacterial species present in the conjunctival sacs of dogs with bacterial conjunctivitis in Wuhan (Hongshan District, Wuchang District, Jiangxia District, and Huangpi District) and their resistance to aminoglycoside antibiotics, samples of conjunctival sac secretions were collected from 56 dogs with bacterial conjunctivitis in various regions of Wuhan. Drug susceptibility testing for aminoglycoside antibiotics was performed on the most commonly isolated gram-positive and gram-negative bacteria. The expression of two aminoglycoside modifying enzyme genes, *aacA-aphD* and *aac (6′)-Ib*, and three 16S rRNA methyltransferase genes, *rmtB*, *rmtE* and *npmA*, were analyzed by PCR. The results showed that a total of 123 bacterial strains were cultured from 56 conjunctival sac secretion samples, with *Staphylococcus* being the most commonly isolated species, followed by *Escherichia*. Among them, 14 strains of *Staphylococcus pseudointermedius* were not resistant to tobramycin, amikacin, gentamicin or neomycin, but the resistance rates to streptomycin and kanamycin were 35.71% and 42.86%, respectively. Among them, 14 *Escherichia coli* strains were not resistant to tobramycin and gentamicin, but they showed high resistance rates to neomycin and kanamycin (both at 50%). The detection rate of the *aacA-aphD* gene in *Staphylococcus pseudointermedius* strains was 100%. The detection rates of the *rmtB* gene and *rmtE* gene in *Escherichia coli* were 85.71% and 28.57%, respectively, while the *aac(6′)-Ib* gene and *npmA* gene were not detected.

## Simple Summary

In order to investigate the types of pathogenic bacteria present in the conjunctival sac secretions of dogs with bacterial conjunctivitis in Wuhan and the antibiotic resistance patterns of the major pathogens to aminoglycoside antibiotics, this study collected samples from 56 dogs diagnosed with bacterial conjunctivitis in Wuhan. Bacterial isolation, identification, and antimicrobial resistance analysis were performed on the conjunctival sac secretions. The results showed that 123 bacterial strains were isolated from the 56 dogs, with *Staphylococcus pseudointermedius* and *Escherichia coli* being the most prevalent pathogens. Drug sensitivity testing and resistance gene detection were performed for these two bacteria. The findings revealed that both *Staphylococcus pseudointermedius* and *Escherichia coli* were sensitive to tobramycin and gentamicin but exhibited high resistance to kanamycin. All *Staphylococcus pseudointermedius* strains carried the *aacA-aphD* gene, while the detection rates of the *rmtB* and *rmtE* genes in *Escherichia coli* were 85.71% and 28.57%, respectively. No *aac(6′)-Ib* or *npmA* genes were detected in *Escherichia coli*. This study contributes to understanding the pathogen distribution in canine bacterial conjunctivitis and their antibiotic resistance patterns, providing data to support the optimization of clinical treatment strategies.

## 1. Introduction

Canine conjunctivitis is an ocular disease caused by a variety of etiological factors [1], including both infectious and non-infectious causes. Infectious conjunctivitis can be caused by bacteria, fungi, and viruses [2,3,4]. The development of bacterial conjunctivitis in dogs is associated with multiple bacterial infections and trauma. Clinically, the primary ocular signs include photophobia, excessive tearing, conjunctival hyperemia, and the discharge of copious secretions from the inner canthus [5]. Common pathogenic bacteria include *Staphylococcus pseudointermedius*, *Streptococcus*, *Pseudomonas aeruginosa* and *Escherichia coli*. *Staphylococcus pseudintermedius* is also frequently isolated in dogs with pyoderma and otitis externa [6,7,8], making it one of the primary pathogens responsible for infectious diseases in animals. *Streptococcus* exhibited a higher isolation rate in corneal ulcers of affected animals in Switzerland [9]. *Pseudomonas aeruginosa* can rapidly destroy the surface cells of the cornea, leading to the development of corneal ulcers [10]. *Escherichia coli* exhibited a high level of multiple drug resistance (MDR) in the study conducted by Jiwoo Park [11]. These bacteria typically invade the dog’s eyes through direct contact, airborne transmission, or environmental contamination, leading to an inflammatory response in the conjunctiva. 

Factors such as the dog’s immune status, living environment, and daily care can significantly affect the incidence of conjunctivitis [5]. In particular, humid and suboptimal environmental conditions facilitate bacterial proliferation, thereby increasing the dog’s susceptibility to infection. Additionally, trauma, foreign body irritation, and certain systemic diseases (e.g., diabetes) may further increase the risk of infection. Therefore, understanding these factors is crucial for the prevention and treatment of bacterial conjunctivitis in dogs [12]. In recent years, Next-Generation Sequencing (NGS) techniques have been widely applied in veterinary medicine [13]. This technology enables the rapid detection of a wide range of pathogens and the characterization of organisms based on their genetic information [14]. However, it does not provide subsequent phenotypic analysis of antibiotic resistance in specific bacterial strains. In clinical practice, veterinarians are especially concerned with the antibiotic resistance phenotype. For bacterial conjunctivitis, veterinarians typically use antibiotic therapy to eliminate pathogenic bacteria [15]. The antibiotic resistance profile plays a crucial role in determining the selection of antibiotics for clinical treatment [16]. Commonly used antibiotics include aminoglycosides, fluoroquinolones, and macrolides. The choice of specific antibiotics is guided by the results of bacterial culture and antimicrobial susceptibility testing [17].

Bacterial conjunctivitis in dogs is a common condition encountered by veterinarians worldwide [18]. The distribution of bacterial species responsible for this condition varies across different regions, including Asia [19,20,21], Europe [9,22], North America [1,5], and South America [23], leading to region-specific therapeutic approaches. In Wuhan, China, veterinarians at several hospitals have used aminoglycoside antibiotics to treat this condition. However, the widespread use of these antibiotics in clinical practice may lead to the emergence of antibiotic-resistant bacterial strains [24]. Several factors contribute to bacterial resistance, including changes in outer membrane permeability, reduced inner membrane transport, and decreased intracellular drug accumulation due to the overexpression of active efflux systems [25]. Additionally, aminoglycoside-modifying enzymes (AMEs) can modify aminoglycoside antibiotics [26]. Mutations or methylation modifications can alter the binding sites and targets of aminoglycoside antibiotics on the 16S rRNA of the bacterial ribosomal 30S subunit [27]. Among these mechanisms, the overexpression of aminoglycoside-modifying enzymes in bacteria is a primary factor for the development of resistance to this class of antibiotics [28]. 

The *aacA-aphD* gene is a key determinant of aminoglycoside-modifying enzymes in *Staphylococcus* genus. Extensive studies have demonstrated that the *aac(6′)-Ib* gene is the most prevalent aminoglycoside-inactivating enzyme gene in *Escherichia coli* [29,30]. In recent years, plasmid-mediated 16S rRNA methyltransferases have been identified in various pathogenic bacteria. These methyltransferases specifically methylate A1408, leading to high-level resistance of pathogens to aminoglycoside antibiotics. Relevant studies have reported the presence of 16S rRNA methyltransferase genes, such as *armA*, *rmtB*, *rmtE*, *rmtF*, and *npmA* in *Escherichia coli* [31].

The primary objective of this study is to investigate the prevalence of bacterial isolates from dogs with bacterial conjunctivitis in Wuhan, China, and to assess the antimicrobial resistance of major Gram-positive and Gram-negative bacteria to aminoglycosides, including dibekacin, kanamycin, neomycin, tobramycin, streptomycin, and gentamicin. Additionally, this study aimed to determine the presence of aminoglycoside-modifying enzyme genes, *aacA-aphD* and *aac(6′)-Ib*, in the most frequently isolated Gram-positive bacterium, *Staphylococcus pseudointermedius*, and the Gram-negative bacterium *Escherichia coli*. Furthermore, the presence of 16S rRNA methyltransferase genes, *rmtB*, *rmtE*, and *npmA*, in *Escherichia coli* isolates were also investigated. The findings of this study are expected to provide valuable insights for clinical veterinary treatment of bacterial conjunctivitis in dogs and contribute to alleviating concerns regarding the rising levels of bacterial resistance.

## 2. Materials and Methods

### 2.1. Animals and Specimen Collection

From March to September 2024, a total of 56 dogs diagnosed with bacterial conjunctivitis were included in this study. The dogs were recruited from various regions of Wuhan, including animal hospitals in Hongshan District and Wuchang District, private dog kennels in Jiangxia District, and the stray animal protection center in Huangpi District. All dogs were diagnosed with bacterial conjunctivitis by qualified veterinarians, and no additional clinical signs or medical history were recorded. Additionally, no antimicrobial drugs were administered prior to sample collection. Informed consent was obtained from the all pet owners prior to participation in the study.

Specimens were collected using sterile cotton swabs. The dry swab was first moistened with sterile physiological saline, and the moistened swab was then used to collect conjunctival sac secretions from the lower eyelid of each dog. The obtained sample was transferred in a 5 mL centrifuge tube containing sterile physiological saline. The samples were stored at 4 °C and transported to the laboratory for bacterial isolation and culture within 24 h.

### 2.2. Chemical and Reagents

LB solid medium, LB liquid medium, and M-H medium were purchased from Haibo Biotechnology Co., Ltd. (Wuhan, China). The 2×Rapid Taq Master Mix enzyme and 10×DNA loading buffer were purchased from Nuoweizan Biotechnology Co., Ltd. (Nanjing, China). The GoldBand DL2000 Marker and YeaRed nucleic acid dye were purchased from Yisheng Biotechnology Co., Ltd. (Shanghai, China). TAE (50×) was purchased from Lanjieke Technology Co., Ltd. (Hefei, Anhui, China). Bacterial genomic DNA extraction kits were purchased from Aidelai Biotechnology Co., Ltd. (Beijing, China). Amikacin, kanamycin, neomycin, tobramycin, streptomycin, and gentamicin were purchased from Hangzhou Microbial Reagent Co., Ltd. (Hangzhou, China). The quality control strains *Escherichia coli* (ATCC 25922) and *Staphylococcus aureus* (ATCC 25923) were purchased from the China Veterinary Drug Supervision Institute.

### 2.3. Bacterial Culture and Identification

The collected samples were inoculated onto LB solid medium using the three-zone streaking method within 24 h and incubated at 37 °C for 24 h to obtain single colonies for isolation and purification. The purified strains were inoculated into LB liquid medium and cultivated with shaking at 37 °C for 18 h at 180 rpm. A 1 mL aliquot of the bacterial suspension was centrifuged, and the pellet was resuspended in 1 mL of ddH_2_O, followed by an additional 100 µL of ddH_2_O. The mixture was then boiled for 10 min, then centrifuged again. The supernatant was transferred to a sterile centrifuge tube to obtain the bacterial genomic DNA.

The extracted bacterial genomic DNA was used as a template for PCR amplification of the target fragment with the universal 16S rRNA primers 27F/1492R (primer sequences: F: 5′-AGAGTTTGATCCTGGCTCAG-3′, R: 5′-TACCTTGTTACGACTT-3′).

The PCR reaction mixture consisted of 25 µL of 2×Rapid Taq Master Mix, 1 µL of bacterial genomic DNA template, 2 µL of each upstream and downstream primer and ddH_2_O added to a final volume of 50 µL. The reaction conditions were as follows: pre-denaturation at 95 °C for 3 min, followed by 35 cycles of denaturation at 95 °C for 15 s, annealing at 50 °C for 15 s, and extension at 72 °C for 20 s. The reaction was completed with a final extension at 72 °C for 5 min to obtain the PCR product.

The PCR products were subjected to electrophoresis with a 1% agarose gel. Electrophoresis was carried out at 150 V and 200 mA for 20 min. After electrophoresis, the agarose gel was imaged using a gel imaging system to check for the presence of the target bands. The PCR products containing target bands in the agarose gel were sent to Wuhan Jinkairui Company for sequencing. The sequencing results were then submitted to the NCBI website, and sequence comparisons were performed using BLAST to determine the taxonomic relationship of the bacteria.

### 2.4. Antimicrobial Susceptibility Test

The resistance of isolated bacteria to six aminoglycoside antibiotics was determined using the K-B method (disc diffusion method). A single colony of the purified bacteria was selected and inoculated into a centrifuge tube containing sterile physiological saline to prepare a bacterial suspension at the 0.5 McFarland standard. A sterile cotton swab, dipped into the bacterial suspension, was then used to inoculate the surface of M-H agar plates. Each M-H agar plate was inoculated with bacterial suspension three times, with each inoculation rotated by 60°. After inoculation, the plates were left to dry at room temperature for 5 min. The antibiotic discs were placed on the surface of the inoculated M-H agar, ensuring that the centers of the discs were ≥24 mm apart and at least 15 mm from the edge of the plate. Six antibiotic discs were placed on a 90 mm plate. After placement, the plates were allowed to stand at room temperature for 15 min before being inverted and incubated in a 37 °C incubator for 16–18 h. Three replicate susceptibility tests were performed for each strain. Finally, the inhibition zones were measured. The interpretation of the antibiotic susceptibility test results was carried out according to the standards set by the Clinical and Laboratory Standards Institute [12]. Quality control strains, *Escherichia coli* (ATCC 25922) and *Staphylococcus aureus* (ATCC 25923), were included.

The antibiotic contents in the antibiotic discs were as follows: tobramycin (TOB), 10 µg; gentamicin (GEN), 10 µg; streptomycin (STR), 10 µg; neomycin (NEO), 30 µg; kanamycin (KAN), 30 µg; and amikacin (AMK), 30 µg.

### 2.5. Detection of Antibiotic Resistance Genes

The antibiotic resistance gene sequences and primers were obtained from NCBI. The primer sequences were synthesized by Wuhan Jinkairui Company (see Table 1). Genomic DNA from major Gram-positive and Gram-negative bacteria was extracted using a bacterial genomic DNA extraction kit. The target gene was amplified by PCR, using the reaction system and conditions as described above, adjusting the annealing temperature based on the Tm values of the primers. The obtained PCR products were analyzed by agarose gel electrophoresis to determine the size of the target product. After electrophoresis, the gel was examined using a gel imaging system to confirm the presence of the target band.

## 3. Results

### 3.1. Bacterial Culture Results of Conjunctival Sac Secretions from Dogs with Bacterial Conjunctivitis in Wuhan

A total of 123 bacteria strains were cultured in 56 samples of conjunctivitis secretions of dogs with bacterial conjunctivitis. The most common pathogenic genus was *Staphylococcus*, with an isolation rate of 28.46%, followed by *Escherichia* at 11.38%. The most frequently isolated pathogens were *Staphylococcus pseudointermedius* and *Escherichia coli*, with 14 strains each, accounting for 10.45% of all bacterial isolates. *Corynebacterium* was isolated 11 strains, with an isolation rate of 8.94%. Among all the isolated bacteria, Gram-positive bacteria accounted for 74 strains (60.16%), while Gram-negative bacteria accounted for 49 strains (39.84%). The isolation rate of cocci was higher among Gram-positive bacteria, while the isolation rate of bacilli was higher among Gram-negative bacteria, which included one strain of *Brucella*. The details of the bacterial culture results are provided in Table 2.

### 3.2. Comparison of Antimicrobial Susceptibility Between Major Gram-Positive Bacteria and Gram-Negative Bacteria

Among the 74 strains of Gram-positive bacteria, *Staphylococcus pseudointermedius* was the most prevalent, with a total of 14 strains. Among the 49 strains of Gram-negative bacteria, *Escherichia coli* was the most prevalent, also with 14 strains. *Staphylococcus pseudointermedius* exhibited low resistance to tobramycin, amikacin, gentamicin, and neomycin, but high resistance to streptomycin and kanamycin. *Escherichia coli* exhibited low resistance to tobramycin and gentamicin, while exhibiting high resistance to neomycin and kanamycin. Both bacteria exhibited 0% resistance to tobramycin and gentamicin. The resistance rates of *Staphylococcus pseudointermedius* and *Escherichia coli* to kanamycin were 42.86% and 50%, respectively. The drug resistance profiles for the six antibiotics are presented in Table 3 and Table 4, and the antibiotic sensitivity results are provided in Table 5.

### 3.3. Detection of Antibiotic Resistance Genes in Major Gram-Positive Bacteria and Gram-Negative Bacteria

The target band of 220 bp was amplified from all 14 strains of *Staphylococcus pseudointermedius*, indicating that all 14 strains contained the *aacA-aphD* gene (Figure S1).

Detection of the *aac(6′)-Ib*, *rmtE, rmtB*, and *npmA* resistance genes in the 14 strains of *Escherichia coli* revealed that some strains amplified target bands of 976 bp or 756 bp, with four strains containing both target bands. These results indicate that 12 strains of *Escherichia coli* contained the *rmtB* gene, with a detection rate of 85.71%. Four strains contained the *rmtE* gene, with a detection rate of 28.57%, while no *aac(6′)-Ib* or *npmA* genes were detected (Figures S2–S5).

## 4. Discussion

The presence of bacteria is not the sole factor contributing to the development of conjunctivitis. In addition to the administration of appropriate antibiotics, it is crucial for veterinarians to focus on enhancing the immune function of affected dogs during treatment [32]. Inadequate treatment or insufficient disease control may facilitate bacterial invasion of the cornea via the conjunctival blood vessels, potentially resulting in keratitis or corneal ulcers [33], and indirectly influencing the development of cataracts and glaucoma [34,35]. Therefore, bacterial conjunctivitis requires timely diagnosis and intervention to prevent further complications. In this study, conjunctival sac secretion samples were collected from 56 dogs with bacterial conjunctivitis across four regions in Wuhan. *Staphylococcus pseudintermedius* was isolated from samples in all regions and identified as one of the most prevalent pathogens. These findings are consistent with those reported by Wang Li, who studied the microbial flora in the conjunctival sacs of dogs with ulcerative keratitis in Beijing, as well as Zhang Hongchao’s report on the bacterial species in 102 cases of canine corneal ulcers [19,36]. The conjunctival surface of normal dogs is not sterile [37], with bacterial cultures testing positive in 39% to 87% of normal dogs [38]. *Staphylococcus pseudointermedius* has been identified in normal dogs and dogs with conjunctivitis or keratitis [39,40]. Therefore, *Staphylococcus pseudointermedius* is considered an opportunistic pathogen in ocular conditions [41]. In this study, *Escherichia coli* was also one of the most frequently isolated pathogens, which contrasts with the findings of George Cosmin Nadăș [42]. This discrepancy may be attributed to differences in geographic location and the nature of the diseases investigated. *Streptococcus* spp., *Pseudomonas* spp., and *Bacillus* spp. are also common genera associated with infectious ocular diseases in animals [23,43,44]. In this study, a total of 2 strains of *Streptococcus* (11.38%), 6 strains of *Pseudomonas* (4.88%), and 11 strains of *Bacillus* (8.94%) were isolated. In addition, one strain of *Brucella* was also isolated in this study. Three cases of canine endophthalmitis caused by *Brucella* infection have been reported internationally [43], with clinical manifestations including chronic or recurrent uveitis, ranging from mild to moderate anterior uveitis, excessive iris pigmentation, significant vitreous infiltration, and multifocal chorioretinitis [45]. The disease is primarily managed through a combination of antibiotic therapy to alleviate symptoms, ultimately aiming for resolution of the condition. It is important to note that *Brucella* can cause infections in humans, presenting with symptoms such as fever, fatigue, diaphoresis, and organ enlargement [46]. Proper protective measures should be taken during diagnosis and treatment.

In this study, the drug sensitivity of 14 strains of *Staphylococcus pseudintermedius* and 14 strains of *Escherichia coli* strains to six aminoglycoside antibiotics was tested. Both groups showed no resistance to tobramycin and gentamicin, while a high resistance rate to kanamycin was observed. In Romania, the antibiotic resistance phenotype of *Staphylococcus pseudointermedius* closely resembles the findings of the present study. Additionally, the present study reported that *Staphylococcus pseudintermedius* isolates from ocular conjunctival sacs exhibited resistance to chloramphenicol, doxycycline, and penicillin [42]. In recent years, an increasing number of *Escherichia coli* strains isolated from ocular infections have shown resistance to ciprofloxacin [47]. This resistance has been linked to mutations in the *gyrA* and *parC* genes, which are associated with enhanced resistance to fluoroquinolones [48]. The results of this study suggest that tobramycin and gentamicin are effective against bacterial conjunctivitis in dogs in the Wuhan area, highlighting the clinical need for effective and targeted antibiotics in veterinary practice. The highest detection rate of drug resistance genes was found in the *aacA-aphD* gene within *Staphylococcus pseudointermedius*, with a detection rate of 100%. This was followed by the *rmtB* gene in *Escherichia coli*, which had a detection rate of 85.71%. The *aac(6′)-Ib* and *npmA* genes were not detected, which differs from the findings reported by Feng Manyu [49]. This discrepancy may be attributed to differences in bacteria sources, as well as variations in the epidemiological distribution of aminoglycoside resistance genes across different regions and seasons. Although research suggests that the antibiotic resistance phenotype of bacteria is determined by their resistance genotype, complete consistency between the resistance phenotype and genotype is not always observed [50]. In this study, no significant correlation was found between the resistance phenotype and genotype of the tested bacteria. This may be due to the influence of mutations in resistance genes on the level of resistance, as well as the potential impact of culture conditions on the antibiotic sensitivity results.

This study investigated antibiotic resistance in aerobic bacteria responsible for bacterial conjunctivitis. In clinical practice, the presentation of this condition requires consideration of anaerobic bacterial infection, as these factors can assist in the identification of the underlying pathogens [51]. Incorporating such considerations into diagnostic protocols can provide valuable insights for treatment strategies. Furthermore, it is crucial to recognize the role of breed in the occurrence and susceptibility to bacterial conjunctivitis [52]. Variations in breed-specific physiological characteristics [53], immune responses, and ocular health may influence predisposition to this condition. For instance, brachycephalic breeds like Pugs and Saint Bernards, which frequently display flattened facial features and wider eyelid openings, can experience incomplete eyelid closure and insufficient tear distribution across the cornea. This contributes to dry eye, infection, and potential conjunctivitis [42,54]. Breeds with larger eyes, including Shih Tzus and Pomeranians, possess more exposed corneal surfaces, increasing their susceptibility to external irritants and bacterial infections, which may lead to conjunctivitis [36,55]. Long-haired breeds, such as Maltese and Poodles, are prone to irritation from eyelashes and hair, which can cause ocular discomfort and predispose them to bacterial infection [56]. A thorough understanding of these breed-related factors, combined with clinical presentations, is essential for optimizing the management and treatment outcomes of bacterial conjunctivitis.

## 5. Conclusions

This study investigated the common bacteria present in the conjunctival sac secretions of dogs with bacterial conjunctivitis in Wuhan (Huangpi District, Wuchang District, Jiangxia District, and Hongshan District), as well as the resistance of major pathogens to aminoglycoside antibiotics. The results showed that the most prevalent Gram-positive bacterium in the conjunctival sac secretions of dogs with bacterial conjunctivitis was *Staphylococcus pseudointermedius*, while the most common Gram-negative bacterium was *Escherichia coli*. The *Staphylococcus pseudointermedius* and *Escherichia coli* strains isolated in this study were not resistant to tobramycin and gentamicin, but showed a high level of resistance to kanamycin. All *Staphylococcus pseudointermedius* strains carried the *aacA-aphD* gene, and the rates of the *rmtB* and *rmtE* genes in *Escherichia coli* were 85.71% and 28.57%, respectively, with no detection of the *aac(6’)-Ib* or *npmA* genes. The analysis of the antibiotic resistance phenotypes and genotypes of these two bacterial species revealed an inconsistency between the resistance phenotypes and genotypes.

## Figures and Tables

**Table 1:** Primer Sequences for Antibiotic Resistance Genes.

Resistance Gene	Primer Sequence	Annealing Temperature	Target Band
*aacA-aphD*	F: 5′-CCAAGAGCAATAAGGGCATA-3′	49.6 °C	220 bp
	R: 5′-CACTATCATAACCACTACCG-3′		
*Aac(6′)-Ib*	F: 5′-TCCCGAGGTCACCAAGAT-3′	50.1 °C	625 bp
	R: 5′-TCGATGGAAGGGTTAGGC-3′		
*rmtE*	F: 5′-GTTCCCGGCAACGTATTA-3′	47.9 °C	976 bp
	R: 5′-ACAGACACGGCATCAAGT-3′		
*rmtB*	F: 5′-CCCCAAACAGGCCGTAGA-3′	52.4 °C	756 bp
	R: 5′-ACATCGCCCGCAGAAAGC-3′		
*npmA*	F: 5′-TAACTCCGCTAATAATGC-3′	43.3 °C	860 bp
	R: 5′-AACCGTCTTTGTTCCTTT-3′		

**Table 2:** Bacterial Culture Results of Conjunctival Secretions in Dogs with Bacterial Conjunctivitis.

Strain Type		Number of Strains (*n*/Strain)	Composition Ratio
Gram-positive Bacteria (*n* = 74, 60.16%)	*Staphylococcus* spp.	35	28.46%
	*Corynebacterium* spp.	11	8.94%
	*Bacillus* spp.	5	4.07%
	*Streptococcus* spp.	2	1.63%
	*Psychrobacter sanguinis* spp.	8	6.50%
	*Brevibacterium* spp.	6	4.88%
	Others	7	5.69%
Gram-negative Bacteria (*n* = 49, 39.84%)	*Escherichia* spp.	14	11.38%
	*Enterobacter* spp.	7	5.69%
	*Chryseobacterium* spp.	5	4.07%
	*Moraxella* spp.	5	4.07%
	*Monas* spp.	7	5.69%
	*Pseudomonas* spp.	6	4.88%
	*Brucella* spp.	1	0.81%
	Others	4	3.25%
Total		123	100%

Note: The bacterial species listed in the table may represent multiple pathogenic bacteria cultured from the same sample in the analysis.

**Table 3:** Antibiotic Resistance of *Staphylococcus pseudointermedius* to Six Antibiotics.

Strain	AMK	KAN	NEO	TOB	STR	GEN
	Sensitivity	Sensitivity	Sensitivity	Sensitivity	Sensitivity	Sensitivity
W1	S	R	I	S	R	S
W2	S	R	S	S	R	I
W3	S	I	S	S	S	S
W4	S	S	S	S	S	S
W5	S	S	S	S	S	S
W6	S	S	S	S	S	S
W7	S	I	S	S	S	S
W8	S	I	S	S	S	S
W9	S	I	S	S	S	S
W10	S	I	S	S	S	S
W11	S	R	S	S	R	I
W12	S	R	S	S	S	I
W13	S	R	S	S	R	S
W14	I	R	S	S	R	S

Note: W1–14: 14 strains of *Staphylococcus pseudointermedius*. S: Sensitive, I: Intermediate, R: Resistant.

**Table 4:** Antibiotic Resistance of *Escherichia coli* to Six Antibiotics.

Strain	AMK	KAN	NEO	TOB	STR	GEN
	Sensitivity	Sensitivity	Sensitivity	Sensitivity	Sensitivity	Sensitivity
D1	S	S	S	S	S	S
D2	S	R	R	S	I	I
D3	S	R	R	S	S	I
D4	I	R	R	S	I	S
D5	I	S	S	S	I	I
D6	S	S	S	S	I	I
D7	I	R	R	S	I	S
D8	I	R	R	S	I	I
D9	R	I	I	S	I	I
D10	R	R	R	I	R	I
D11	I	S	S	S	I	S
D12	I	I	S	S	I	I
D13	I	R	R	S	I	I
D14	I	I	I	S	I	I

Note: D1–14: 14 strains of *Escherichia coli*. S: Sensitive, I: Intermediate, R: Resistant.

**Table 5:** Antimicrobial Susceptibility Test Results for *Staphylococcus pseudointermedius* and *Escherichia coli*.

Antimicrobial Drugs	*Escherichia coli* (*n* = 14)		*Staphylococcus pseudointermedius* (*n* = 14)	
	Drug-Resistant Strains	Resistance Rate	Drug-Resistant Strains	Resistance Rate
AMK	2	14.29%	0	0.00%
KAN	7	50.00%	6	42.86%
TOB	0	0.00%	0	0.00%
STR	1	7.14%	5	35.71%
GEN	0	0.00%	0	0.00%
NEO	7	50.00%	0	0.00%

## Data Availability

The data presented in this study are available on request from the corresponding author. The data are not publicly available due to the institutional privacy policy.

## Supplementary Materials

The following supporting information can be downloaded at: https://www.mdpi.com/article/10.3390/vetsci12010021/s1, Figure S1: Agarose Gel Electrophoresis Results of the *aacA-aphD* Antibiotic Resistance Gene. W1–14: 14 strains of *Staphylococcus Pseudintermedius*; M: DL2000 Marker; N: negative control; Figure S2: Agarose Gel Electrophoresis Results of the *rmtB* Antibiotic Resistance Gene. D1–14: 14 strains of *Escherichia coli*; M: DL1000 Marker; N: negative control; Figure S3: Agarose Gel Electrophoresis Results of the *rmtE* Antibiotic Resistance Gene. D1–14: 14 strains of *Escherichia coli*; M: DL1000 Marker; N: negative control; Figure S4: Agarose Gel Electrophoresis Results of the *npmA* Antibiotic Resistance Gene. D1–14: 14 strains of *Escherichia coli*; M: DL2000 Marker; N: negative control; Figure S5: Agarose Gel Electrophoresis Results of the *aac(6′)-Ib* Antibiotic Resistance Gene. D1–14: 14 strains of *Escherichia coli*; M: DL2000 Marker; N: negative control.
