# Supplementary material for: Isolation, Identification, and Antimicrobial Susceptibilities of Bacteria from the Conjunctival Sacs of Dogs with Bacterial Conjunctivitis in Different Regions of Wuhan, China
Source: Vet Sci. 2025 Jan 6;12(1):21. doi: 10.3390/vetsci12010021 (PMC11769483; doi:10.3390/vetsci12010021)
Supplement: Supplementary file 1 [file vetsci-12-00021-s001.zip › vetsci-3355961-supplementary.pdf]

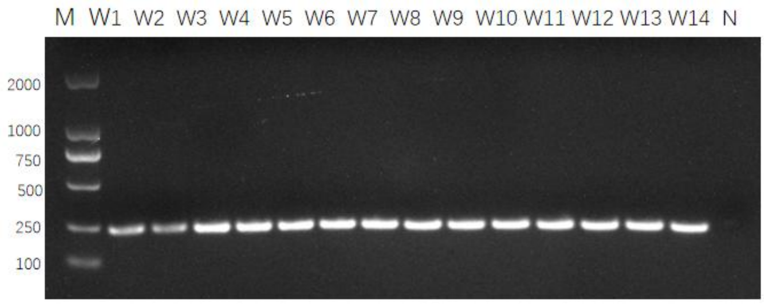


Figure S1: Agarose Gel Electrophoresis Results of the *aacA-aphD* Antibiotic Resistance Gene. W1-14: 14 strains of *Staphylococcus Pseudintermedius*; M: DL2000Marker; N: negative control

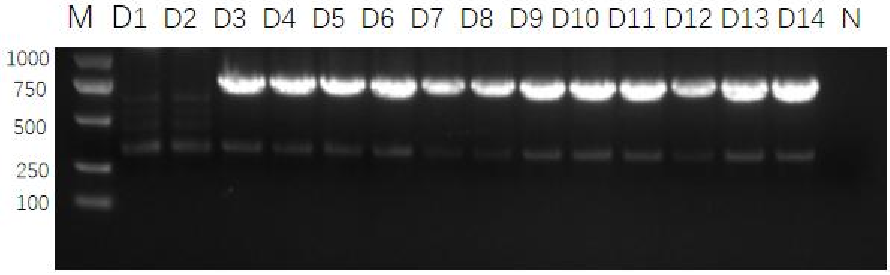


Figure S2: Agarose Gel Electrophoresis Results of the *rmtB* Antibiotic Resistance Gene. D1-14: 14 strains of *Escherichia coli*; M: DL1000 Marker; N: negative control

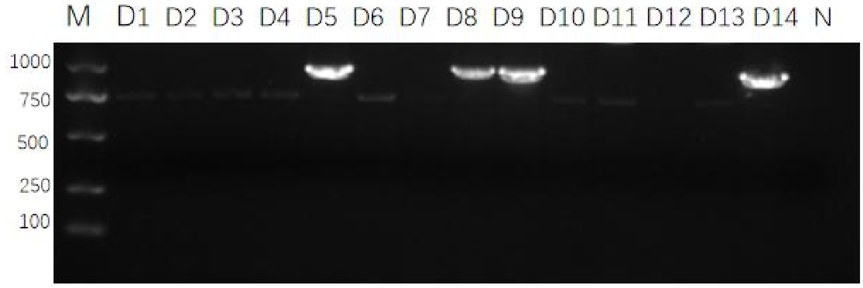


Figure S3: Agarose Gel Electrophoresis Results of the *rmtE* Antibiotic Resistance Gene. D1-14: 14 strains of *Escherichia coli*; M: DL1000 Marker; N: negative control

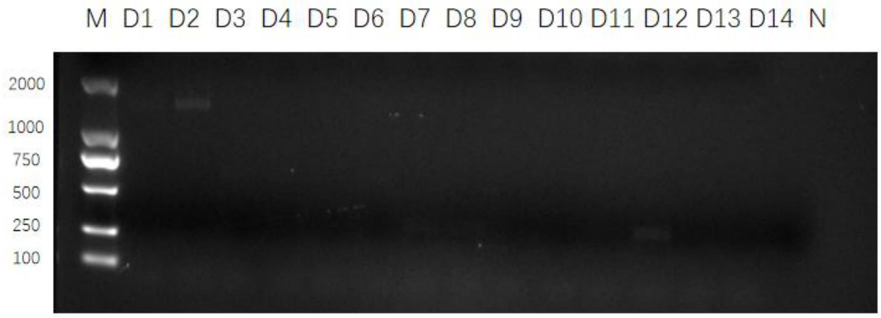


Figure S4: Agarose Gel Electrophoresis Results of the *npmA* Antibiotic Resistance Gene. D1-14: 14 strains of *Escherichia coli*; M: DL2000 Marker;N: negative control

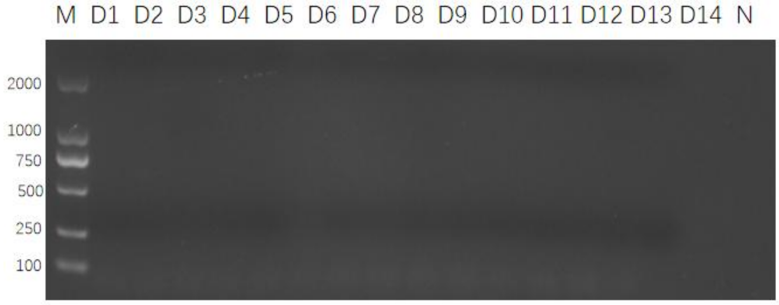


Figure S5: Agarose Gel Electrophoresis Results of the *aac(6')-Ib* Antibiotic Resistance Gene. D1-14: 14 strains of *Escherichia coli*; M: DL2000 Marker; N: negative control
